# 489. SARS-CoV-2 Seroprevalence and Antibody Response Among Pregnant People in Seattle, WA

**DOI:** 10.1093/ofid/ofab466.688

**Published:** 2021-12-04

**Authors:** Sylvia LaCourse, Alisa Kachikis, Kelsey L Kinderknecht, Romeo R Galang, Lauren B Zapata, Krissy M Yamamoto, Carol C Salerno, Alexander L Greninger, Janet A Englund, Alison Drake

**Affiliations:** 1 Medicine & Global Health, University of Washington, Seattle, Washington; 2 University of Washington, Seattle, Washington; 3 U.S. Centers for Disease Control and Prevention, Atlanta, Georgia; 4 US Centers for Disease Control and Prevention, Buford, Georgia; 5 Valley Medical Center, UW, Seattle, Washington; 6 Seattle Children’s Hospital/Univ. of Washington, Seattle, Washington

## Abstract

**Background:**

Antenatal care is a unique opportunity to assess SARS-CoV-2 seroprevalence and antibody response in pregnant people, including those with previously unknown infection.

**Methods:**

Pregnant people were screened for SARS-CoV-2 IgG during antenatal care or delivery in Seattle, Washington with Abbott Architect chemiluminescent immunoassay which provides quantitative index (positive ≥1.4). Participants with IgG+ results or identified with RT-PCR+ results via medical records were invited to enroll in a longitudinal evaluation of antibody responses. We report preliminary results of an ongoing seroprevalence and longitudinal study with planned 18-month follow-up.

**Results:**

Between September 9, 2020–May 7, 2021, we screened 1304 pregnant people; 62 (4.8%) tested SARS-CoV-2 IgG+, including 28 (45%) with known prior SARS-CoV-2 infection. Among participants testing IgG+, median age was 32 years (interquartile range [IQR] 26–35) and median gestational age was 21 weeks (IQR 12–38) at screening; median IgG index was 3.2 (IQR 2.1–4.9, range 1.4–9.9), including 3.9 (IQR 2.3–5.8) among those with vs. 2.7 (IQR 1.9–4.2) among those without prior RT-PCR+ results (p=0.05 by Wilcoxon rank-sum). Of 30 longitudinal study participants enrolled, 24 tested IgG+ at baseline (75% with prior RT-PCR+ result) and 6 tested IgG- on enrollment but were identified as previously RT-PCR+ via medical records; 24/30 (80%) reported previous symptoms. Of 24 participants testing IgG+ at baseline, 14 (58%) had first follow-up IgG results at median of 66 days (IQR 42–104) since initial testing, with median IgG index of 2.0 (IQR 1.0–3.8). 9/14 (64%) participants with repeat IgG testing remained IgG+ at first follow-up (≤280 days after first RT-PCR+ result for those with and ≥104 days after first IgG detection for those without prior RT-PCR+ results), while 5/14 (26%) had a negative Abbott IgG test at a median of 81 days (IQR 75–112) since initial testing.

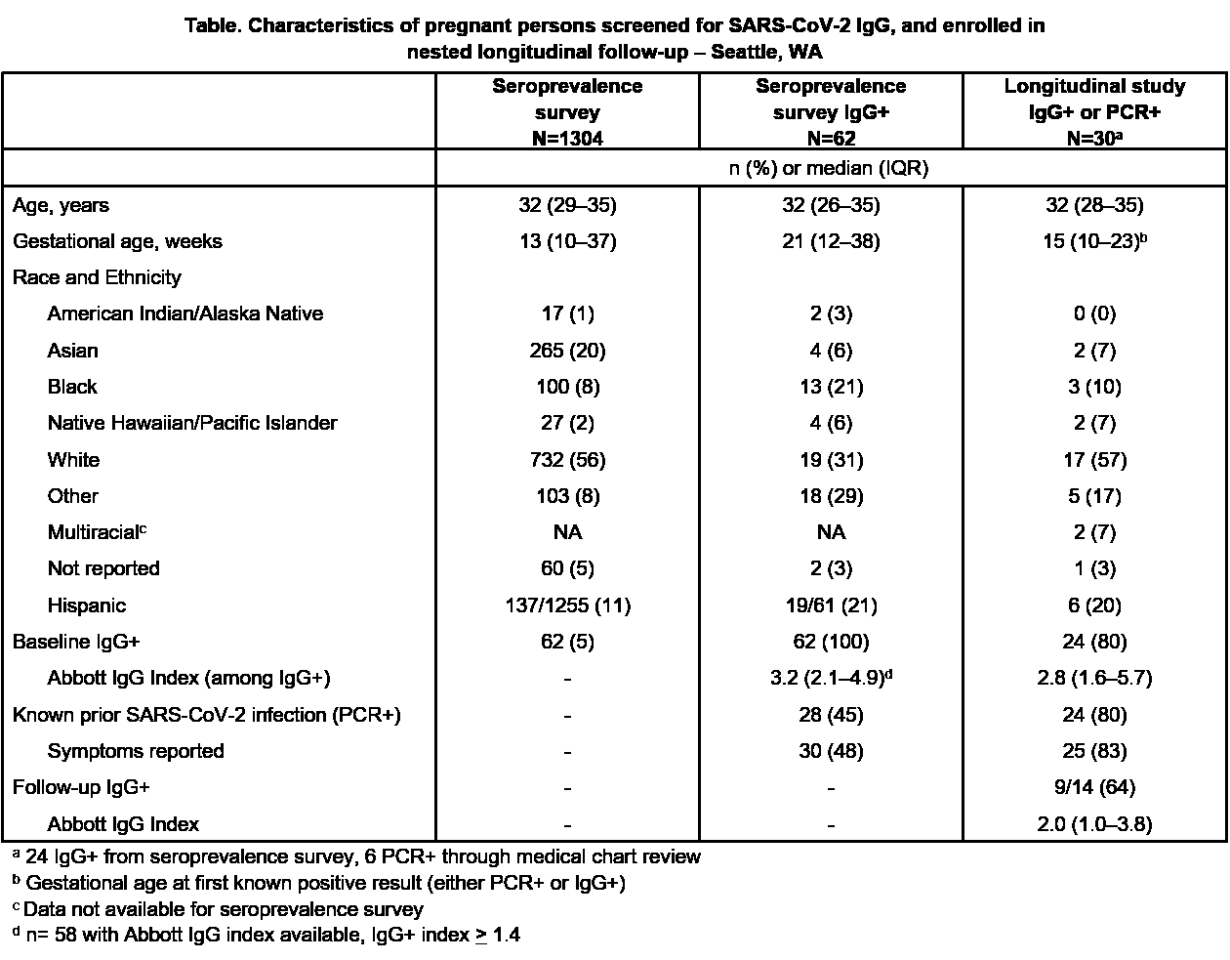

**Conclusion:**

Nearly half of pregnant people testing SARS-CoV-2 IgG+ reported no known prior SARS-CoV-2 diagnosis or symptoms. SARS-CoV-2 IgG antibody response and durability in pregnancy has implications for maternal and neonatal protection and susceptibility and highlights potential benefits of vaccination in this population.

**Disclosures:**

**Sylvia LaCourse, MD**, **Merck** (Grant/Research Support) **Alisa Kachikis, MD, MS**, **GlaxoSmithKline** (Consultant)**Pfizer** (Consultant) **Alexander L. Greninger, MD, PhD**, **Abbott** (Grant/Research Support)**Gilead** (Grant/Research Support)**Merck** (Grant/Research Support) **Janet A. Englund, MD**, **AstraZeneca** (Consultant, Grant/Research Support)**GlaxoSmithKline** (Research Grant or Support)**Meissa Vaccines** (Consultant)**Pfizer** (Research Grant or Support)**Sanofi Pasteur** (Consultant)**Teva Pharmaceuticals** (Consultant) **Alison Drake, PhD, MPH**, **Merck** (Grant/Research Support)

